# Management of Patient Care in Hemodialysis While Focusing on Cardiovascular Disease Events and the Atypical Role of Hyper- and/or Hypotension: A Systematic Review

**DOI:** 10.1155/2016/9710965

**Published:** 2016-10-19

**Authors:** Amjad Khan, Amer Hayat Khan, Azreen Syazril Adnan, Syed Azhar Syed Sulaiman, Siew Hua Gan, Irfanullah Khan

**Affiliations:** ^1^Discipline of Clinical Pharmacy, School of Pharmaceutical Sciences, Universiti Sains Malaysia, 11800 Penang, Malaysia; ^2^Chronic Kidney Disease Resource Centre, School of Medical Sciences, Universiti Sains Malaysia, Health Campus, 16150 Kubang Kerian, Kelantan, Malaysia; ^3^Department of Biotechnology, Quaid-i-Azam University, Islamabad 45320, Pakistan; ^4^Human Genome Centre, School of Medical Sciences, Health Campus, Universiti Sains Malaysia, Kubang Kerian, Kelantan, Malaysia

## Abstract

*Background*. Hemodialysis related hemodynamic instability is a major but an underestimated issue. Moreover, cardiovascular events are the leading cause of morbidity and mortality associated with blood pressure in hemodialysis patients. However, there have been many controversies regarding the role and management of hyper- and/or hypotension during hemodialysis that needs to be addressed.* Objective*. To critically review the available published data on the atypical role of hyper- and/or hypotension in cardiovascular associated morbidity and mortality in patients on hemodialysis and to understand the discrepancies in this context.* Methods*. A comprehensive search of literature employing electronic as well as manual sources and screening 2783 papers published between Jan 1980 and Oct 2015 was conducted to collect, identify, and analyze relevant information through peer-reviewed research articles, systematic reviews, and other published works. The cardiovascular events, including accelerated atherosclerotic cardiovascular disease (ASCVD), stroke, heart failure, myocardial infarction, myocardial ischemia, and stress induced myocardial dysfunction, leading to death were considered relevant.* Results*. A total of 23 published articles met the inclusion criteria and were included for in-depth review and analysis to finalize a comprehensive systematic review article. All the studies showed a significant association between the blood pressure and cardiovascular disease events in hemodialysis patients.* Conclusions*. Both intradialytic hypertension/hypotension episodes are major risk factors for cardiovascular mortality with a high percentage of probable causality; however, clinicians are faced with a dilemma on how to evaluate blood pressure and treat this condition.

## 1. Introduction

In chronic kidney disease (CKD) the kidney function is gradually reduced over a period of time. As a result, glomerular filtration rate (GFR) of the patient drops to <60 mL/minute and the albumin-to-creatinine ratio of patient's urine becomes >30 mg/g. The occurrence of a total, irreversible, and permanent kidneys' failure is termed as end-stage renal disease (ESRD) where fluid is retained by the patient's body resulting in the accumulation of harmful wastes. Consequently, the ESRD patients need suitable treatment in order to replace and compensate for the work of such failed kidneys.

The prevalence of CKD and ESRD is increasing worldwide. According to the US National Kidney Foundation, 26 million American adults suffer from CKD, the precursor to ESRD, and millions more are at increased risk [1]. It has been estimated that more than 700,000 Americans have ESRD [2]. In Malaysia, increase in ESRD was largely driven by the increasing incidence of diabetic kidney disease accounting for 58% of new patients accepted for dialysis [3]. Similarly, CKD is rapidly growing in Pakistan due to increasing number of patients with diabetes and high blood pressure, late diagnosis, and high volume of kidney stone disease leading to death of about 20,000 Pakistanis every year [4].

Cardiovascular disease is the main cause of death in patients with ESRD [5, 6]. It is estimated that ESRD patients are 5 to 20 times more likely to die because of cardiovascular causes than the general population [7–9]. It is also a well-established fact that blood pressure plays a decisive role in CVD based mortality and morbidity in ESRD patients on hemodialysis; however, a careful literature review of various observational studies is more often demonstrating highly conflicting and controversial data regarding the relationships between blood pressure (hyper- and hypotension) and CVD based mortality in patients on hemodialysis [10–17].

Zager et al. described a “U”-shaped relationship between postdialysis systolic blood pressure (SBP) and mortality [17] and in another study by Port et al. [18] it was found that predialysis SBP 110 mmHg was associated with increased mortality. On the other hand, investigators of another study did not observe any association between BP and mortality [19]. In addition to the above, Park et al. [20] described the relationship of dialysis alterations (before, during, and after the process of dialysis) with changes in BP and mortality. These authors also compared outcomes across strata of predialysis SBP, ultrafiltration rates, and time on dialysis, none of which significantly modified the primary findings [20].

Kalantar-Zadeh and his coworker [21–23] presented the theory of “*reverse epidemiology*” which states that “conventional risk factors of cardiovascular disease and mortality in the general population such as body mass, serum cholesterol, and blood pressure are also found to relate to outcome in maintenance dialysis patients, but often in an opposite direction.”

Chao et al. reported that safe and smooth achievement of ultrafiltration and solute removal goal in chronic dialysis patients is prohibited by a decrease of systolic or mean blood pressure (intradialytic hypotension) to a certain level. They have referred to various studies elucidating the potential mechanisms involved in the development of intradialytic hypotension, including excessive ultrafiltration and loss of compensatory mechanisms for blood pressure maintenance [24]. However, Charra et al. argue that reverse epidemiology has misleading relevance on dialysis management. According to them the high early mortality generally associated with and attributed to intradialytic hypotension values does not contradict the need of obtaining normal blood pressure in patients on hemodialysis in order to reduce long-term cardiovascular mortality. They, however, suggest for further investigation candidly confirming the ultimate noxious/beneficial role played by antihypertensive medications in patients on hemodialysis [25].

The aim of the current systemic review is to summarize and critically review the available published data related to the atypical role of hyper- and/or hypotension in cardiovascular associated morbidity and mortality in patients on hemodialysis, to understand the discrepancies in this context, and to, possibly, propose suitable strategies for management of patient care with special reference to blood pressure causing cardiovascular events leading to death in hemodialysis patients.

## 2. Methodology

### 2.1. Administrative Information

The protocols and checklist of PRISMA [26] and PRISMA-P 2015 [27, 28] have been followed in writing of this systemic review article. The title of the review is finalized in accordance to the said protocols. The review has no registration number as it has not been registered in registry. All authors contributed to the search, identification, screening, selection, and developing inclusion or exclusion criteria. As there existed wide variability in study populations and also due to different methods adopted for the measuring outcomes, we did not conduct any meta-analysis and the articles were therefore analyzed descriptively with an emphasis on trends. All authors critically reviewed, provided feedback on, and approved the final version of the manuscript to be submitted for publication.

A comprehensive search of literature employing electronic as well as manual sources, published between Jan 1980 and Oct 2015, was conducted in order to collect, identify, and analyze relevant information and knowledge existing in the instant area of our search through relevant peer-reviewed research articles, systematic reviews, and other published works related to blood pressure and cardiovascular events, such as stroke, myocardial infarction, and heart failure, in hemodialysis patients.

### 2.2. Search Strategies

A strategic, systematic, and comprehensive way of search techniques was applied, by the first author in independent capacity, to extract relevant articles of interest employing electronic database system such as PubMed, International Pharmaceutical Abstracts (IPB), Cumulative Index of Nursing and Allied Health Literature (CINAHL), Web of Science, Scopus, Current Content, Embase, All Evidence-Based Medicine (EBM) Reviews (Cochrane DSR, ACP Journal Club, DARE, and CCTR), and Google Scholar, using the Boolean operators for combinations of key words and terms relevant to the topic of this review article, such as patient care management in hemodialysis, cardiovascular morbidity and mortality in hemodialysis, management of cardiovascular events in hemodialysis patients, management of cardiovascular events and blood pressure in hemodialysis patients, the role of hypertension in cardiovascular associated morbidity and mortality in hemodialysis, the role of hypotension in cardiovascular associated morbidity and mortality in hemodialysis, the controversial role of hyper- and/or hypotension in cardiovascular events in hemodialysis patients, end-stage renal disease and hemodialysis, end-stage renal failure and hemodialysis, stage 5 CKD and hemodialysis, advanced CKD, and hemodialysis. Keeping in view the difficulty in translating our specific search questions into concise key word or search terms, all other possible strategies were also exhausted in order to capture all that relevant information that might have been skipped in the database searches. Moreover, the bibliographies of selected articles were reviewed for the said purpose. In addition to the above-mentioned search using electronic database systems, manual sources were also exploited for the said purpose. Similarly, several academic researchers and professional nephrologists were contacted for help. For getting access to full-text research/review articles the library and E-journals facility of University Sains Malaysia (USM) were used. In addition to these, the Higher Education Commission (HEC) sponsored digital library facility of Quaid-i-Azam University (QAU), Islamabad, was also utilized as alumni of QAU. Finally, abstracts and proceedings of several important meetings, conferences, workshops, and seminars organized by various renal societies of nephrology and other relevant bodies or individuals were also reviewed to get the most in this context.

### 2.3. Study Selection

The articles were identified by the principal investigator/first author, independently, through all the electronic sources. After removal of the duplicates, the data were subjected to a two-staged screening process comprised initially of screening by titles followed by abstract screening. This process screened out a major fraction of the initially identified articles from the electronic database systems to be excluded, leading to a smaller sample of leftover articles being qualifying for the 3rd stage of full-text screening process. Eligibility assessment was conducted by Amjad Khan and Amer Hayat independently in an unblinded standardized manner. Disagreements between reviewers were resolved by consensus.

### 2.4. Inclusion and Exclusion Criteria

**Figure 1:**
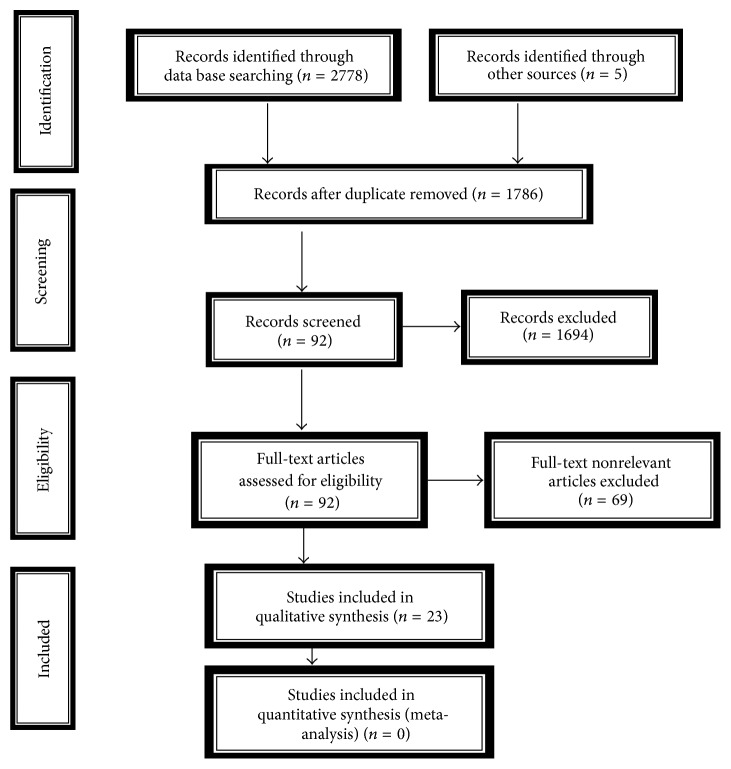
PRISMA flow diagram showing the search strategy and results.

For the purpose of this systemic review, the search was limited to be conducted within a specific period of publication (Jan 1980 to Oct 2015), including the studies with a minimum of 100 participants, published in English, and containing appropriate information and knowledge available in the area of our search question through relevant peer-reviewed research articles, systematic reviews, and other published works related to hemodialysis patients. All other studies lying outside the above narrated range and purview specified for this systematic review were excluded. The screening process has been summarized through PRISMA flow diagram as illustrated in Figure 1.

### 2.5. Data Extraction and Quality Assessment

To assess the study quality, a data extraction sheet was developed and refined after testing on ten randomly selected studies. Amjad Khan extracted the data from included studies and Amer Hayat checked the extracted data. Disagreements were planned to be resolved by mutual discussions; however, any such disagreement did not arise.

## 3. Results

A total of 2783 articles were identified by the principal investigator/first author, independently, through all the electronic sources; out of these articles, the electronic database systems could retrieve 2778 and the Google Scholar searching as 05 (*n* = 2778 + 5 = 2783). After removal of the duplicates (*n* = 997), the data (*n* = 1786) were subjected to a two-staged screening process comprised initially of screening by titles followed by abstract screening. This process screened out a major fraction of the initially identified articles from the electronic database systems to be excluded (*n* = 1694) leading to a smaller sample of leftover articles (*n* = 92) qualifying for the 3rd stage of full-text screening process. After screening of the 92 full-text articles, it was found that only 23 articles (Table 1) were relevant to meet inclusion criteria and were declared successful to be part of further studies to be carried out over them towards the completion of this review. The screening process has been summarized through PRISMA flow diagram as illustrated in Figure 1. There were multiple reasons which led to the exclusion of nonrelevant articles.

All the studies reviewed showed a significant association between the blood pressure and cardiovascular disease events. In one of the articles (s # 8, Table 1), the authors reported that both increases and decreases in blood pressure from before to after hemodialysis would be associated with all-cause and cardiovascular mortality, independent of predialysis BP levels. They identified a U-shaped relationship between pre- and postdialysis changes in BP and all-cause and cardiovascular mortality.

In the instant case, it was found that various observational studies are demonstrating conflicting and controversial data regarding the relationships between blood pressure (hyper- and/or hypotension) and mortality caused by cardiovascular events in patients on hemodialysis. Moreover, these studies have also intensively evaluated relationship between different types of BP measurements and CVD based mortality in hemodialysis patients. Therefore, it was decided to divide and report the findings of this study into various parts as described below.

### 3.1. Relation between BP and CVD Based Mortality

#### 3.1.1. Risk Related to Hypertension Episodes

Among the 23 articles included for qualitative synthesis (Table 1), 12 articles (serial # 1, 4, 5, 6, 10, 11, 12, 13, 14, 19, 20, and 22) focus on and suggest a strong association of hypertension with various cardiovascular disease events leading to morbidity and mortality of CKD or ESRD patients on hemodialysis.

#### 3.1.2. Risk Related to Hypotension Episodes

Only one article (s # 3) reports the strong association of hypotension, measured either predialysis or postdialysis, with increased mortality in patients on hemodialysis.

#### 3.1.3. Risk Related to Both Hyper- and Hypotension Episodes

Among the 23 reviewed articles, 10 studies (s # 2, 7, 8, 9, 15, 17, 18, 21, and 23) reported that both hyper- and hypotension are found to be associated with cardiovascular events based morbidity and mortality in patients on hemodialysis.

### 3.2. Relation between the Type of BP Measurement and CVD Based Mortality

In 18 (s # 1–6, 8, 10–15, and 19–23) out of the 23 articles reviewed, the blood pressure was measured at standardized hemodialysis units, that is, dialysis centers (predialysis, postdialysis, and intradialysis), while the remaining 5 studies (s # 7, 9, 16, 17, and 18) have discussed the blood pressure measurements obtained at home, either by the patient or using an ambulatory blood pressure monitor, and their relationship with CV morbidity and mortality.

## 4. Discussion and Interpretations

Management of patient care in hemodialysis patients is an important but complex task on the part of healthcare team members. In this connection, the randomized controlled trials conducted for reducing primary outcomes of cardiovascular disease events, mortality, and morbidity in such patients have so far not been proven very successful. The area of major concern in the management of hemodialysis patients as related to the above referred outcomes includes the cardiovascular disease burden, control of hyper- and/or hypotension, anemia, inflammation, and abnormalities in mineral metabolism. At times, these issues are further complicated by conflicting and controversial data obtained from observational and randomized controlled trials. The main reason for such controversies could be the limited treatment options for reducing these endpoints in maintenance hemodialysis patients. Therefore, for continuous management of advanced care in patient on hemodialysis it is essential to conduct, in future, well-planned randomized controlled trials in order to meet the goal of improving outcomes. Such trials should consider new therapies to better target these factors, consideration of additional risk factors that have not been well tested to date, and therapies with new targets.

In the light of the literature studied, it is now well established that during hemodialysis treatment the changes occurring in hemodynamics act as a marker for higher risk of patient morbidity and mortality. The most common clinical problems include developments and management of hypertension or hypotension.

It has been reported that accelerated atherosclerotic cardiovascular disease (ASCVD) has close association with high blood pressure [10] and that ASCVD morbidity [11] and mortality [10, 12] may be decreased significantly through successful treatment of hypertension. The defined hypertension among adults should be 140/90 mmHg (systolic BP (SBP)/diastolic BP (DBP)) while in patients with existing ASCVD the recommended target BP is 130/80 mmHg [10]. Recently, a target BP 130/80 mmHg for patients with chronic kidney disease stages I to IV [14] has been recommended. Very recently, the pre-HD (140/90 mmHg) and post-HD (130/80 mmHg) BP targets have also been recommended [15]. However, this recommendation was graded as less authentic and weak because its evidence was extrapolated from the results obtained from general population.

In contrast, another study showed that a blood pressure of 140/90 was associated with a decreased risk for left ventricular hypertrophy but an increased mortality risk [16]. According to the finding of Diaz et al. [29] (Table 1, article at s # 2), amongst the patients on hemodialysis approximately 20% of the patients are prone to episodes of hypotension; and occurrence of intradialytic hypertension is common in approximately 15% of the hemodialysis patients. Hence the results of studies reviewed (Table 1) demonstrated that the relationship of cardiovascular outcomes with hyper- and/or hypotension has been very much controversial. These controversies are mostly related to the particular relationship of blood pressure measurement time and techniques (measurement of predialytic, postdialytic, or intradialytic blood pressure or interdialytic home and ambulatory blood pressure) to cardiovascular morbidity and mortality.

Apart from these, some investigators found and reported a “U”-shaped relationship between postdialysis systolic blood pressure (SBP) and mortality [17]. According to this study mild to moderate elevations in predialysis SBP were not associated with significant increases in ASCVD and all-cause (AC) mortality. Later on similar results were claimed by some other researchers too who reported high postdialysis SBP and low pre- and postdialysis DBP that were associated with increased mortality of the HD patients [13] and in another study by Port et al. [18] it was found that predialysis SBP 110 mmHg was associated with increased mortality. On the other hand, investigators of another study did not observe any association between BP and mortality [19].

In addition to the above, Park et al. [20] described the relationship of dialysis alterations (before, during, and after the process of dialysis) with changes in BP and mortality. They hypothesized another U-shaped relationship between peridialytic hypertension and hypotension and stated that both increases and decreases in BP from before to after dialysis would be associated with all-cause and cardiovascular mortality, independent of predialysis BP levels [20]. The authors found that* modest intradialysis declines* in systolic BP (SBP) (−30 to 0 mm Hg) were associated with the best survival, whereas a pre- to postdialysis decline of less than −30 mm Hg and any pre- to postdialysis increase of more than zero mm Hg were associated with higher mortality. The greatest survival was associated with a pre- to postdialysis change in SBP of −14 mm Hg. They observed similar results for diastolic BP, too.

The controversial theory of “*reverse epidemiology*” [21–23] reported a paradoxical association between hypertension and mortality in hemodialysis patients. It claims that a normal to low blood pressure is associated with poor outcomes, whereas high pressure confers survival advantages, a phenomenon referred to as “reverse epidemiology.” It may be stated that although the above-mentioned paradoxical associations between hypotension and mortality might not be causal, yet the reverse epidemiology of systolic hypertension (HTN) and its relative survival advantage in maintenance hemodialysis (MHD) and chronic heart failure (CHF) patients or other similar populations might have significant clinical and public health implications.

As described above in the results section, the authors of some studies have attributed the cardiovascular associated morbidity and mortality to the hypertension while others have related the same to hypotension in case of patients on hemodialysis. In fact high blood pressures measured either before or after dialysis are either not associated or minimally associated with increased mortality. In addition to this, the association of low BP and cardiovascular related mortality in hemodialysis is further magnified when BP is considered as a time-dependent covariate. Moreover, the phenomenon of lower BP being associated with increased morbidity and mortality in hemodialysis patients has been referred to as* “reverse epidemiology”* of hypertension, which has logically raised concerns of the patients and practitioners regarding lowering of BP among hypertensive patients on hemodialysis.

These facts clearly indicate that proper control of blood pressure is critical in dialysis patients and its importance can never be overemphasized because the risk of other comorbidities will increase under such extreme changes. Therefore, proper knowledge and awareness is required against the effect of such abnormalities and to help in preventing their complications and recurrence [29].

## 5. Limitations

The major limitation of the instant review could be attributed to the possible but inadvertent selection biasness of the retrieved articles, although all possible efforts were made to ensure the inclusion of all relevant and exclusion of all the irrelevant articles. Accordingly, a total of 2783 articles could be retrieved from the databases; however, after removal of the duplicates (*n* = 997), only 1786 remained for screening and further processing and finally only 23 could meet the inclusion criteria. Moreover, we could not conduct any meta-analysis and the articles were therefore analyzed descriptively with an emphasis on trends because of wide variability in study populations and also due to different methods adopted for the measuring outcomes. In addition to the above, while conducting such studies, a judicial analysis and careful assessment of the baseline comorbidities are of utmost importance because the relationship between BP and mortality is stronger in diabetics than in nondiabetics [30]. However, in the instant case no formal assessment of the comorbidities was conducted. It is also worth mentioning over here that the use of antihypertensive agents by the HD patients may significantly affect the risk of CVD based mortality; consequently such type of confounding factors might affect the overall relationship between BP and CVD based mortality. Among the 23 studies included in the instant review, six studies have not reported the use of any such antihypertensive agents and this could be considered a significant factor and important limitation affecting the proposed BP relationship with CVD based mortality.

## 6. Recommendations

Based on the critical review of the shortlisted articles (Table 1), it was revealed that there were a lot of controversies regarding the role and/or management of blood pressure in patients on hemodialysis, for example, uncertainty about how to measure BP in hemodialysis patients, a poor understanding of the association between hyper- and/or hypotension and cardiovascular disease events and other risks of adverse outcomes, and a complex interplay of multiple factors affecting both hypo- and hypertension (systolic and diastolic pressure), so it would be premature to make any specific recommendations regarding BP management in ESRD patients on hemodialysis.

However, it is hereby recommended that in-depth studies are required to further explore the complex physiological and dialysis-related mechanisms influencing BP and also the yet poorly understood association between hyper- and/or hypotension and cardiovascular disease events for better management of BP in patient population on hemodialysis while focusing on both generally applicable plans and individualization in order to determine the BP target and the treatment regimen.

## 7. Conclusions

The study of reviewed articles showed that both hypertension and hypotension episodes are major risk factors for mortality with a high percentage of probable causality. The clinicians are faced with a dilemma on how to evaluate blood pressure and treat this condition. Hypertension is common, difficult to diagnose, and poorly controlled among patients with ESRD. Patients with CKD and regular hemodialysis who experience moderate or severe intradialytic hypotension have significantly higher prevalence of myocardial ischemia and stress induced myocardial dysfunction than those who experience no or mild intradialytic hypotension. It is well established that there exists a relationship between pre- to postdialysis changes in BP and mortality. Both increases and decreases in BP from before to after dialysis would be associated with all-cause and cardiovascular mortality, independent of predialysis BP levels. Topics of major concern in the management of maintenance hemodialysis patients include the overall cardiovascular disease burden, blood pressure control, anemia, abnormalities in mineral metabolism, and inflammation. Overall, treatment options for reducing these endpoints in maintenance hemodialysis patients are limited, and future randomized controlled trials are essential to continuing to advance care in this population, with the goal of ultimately improving hard outcomes. Future trials should consider new therapies to better target these factors, additional risk factors that have not been well tested to date, and therapies with new targets, including inflammation. Further studies with larger numbers could be more conclusive.

## Figures and Tables

**Table 1:** Conclusive summary of the full-text articles included in qualitative synthesis (*n* = 23).

S #	Reference	Title	Conclusion
1	Gorsane et al. 2015 [31]	“Prevalence and Risk Factors of Hypertension in Hemodialysis”	The reduction of BP allows a lower risk of CV events and mortality in HTN patients.

2	Diaz et al. 2015 [29]	“Mortality and Intradialytic Hypertension and Hypotension Episodes”	Intradialytic hypertension/hypotension episodes are major risk factors for mortality with a high percentage of probable causality. The use of beta blockers and calcium channel blockers may confer protection in avoiding increased mortality. Finally the development of cardiomegaly is increased significantly in these patients and may lead to cardiac events.

3	Chao et al. 2015 [24]	“Intradialytic Hypotension and Cardiac Remodeling: A Vicious Cycle”	A bidirectional relationship might exist between intradialytic hypotension and left ventricular hypertrophy in chronic dialysis patients. A more complete understanding of the complex interactions in between could assist the readers in formulating potential solutions for the reduction of both phenomena.

4	Iseki 2015 [32]	“Control of Hypertension and Survival in Hemodialysis Patients”	HTN is risk and must be controlled.

5	Enam et al. 2014 [33]	“Management of Hypertension in the Hemodialysis Population: A Review of the Literature”	Multiple management modalities may benefit.

6	Agarwal et al. 2014 [34]	“Assessment and Management of Hypertension in Patients on Dialysis”	Often medication directed approaches are used; however, no pharmacologic and dialytic approaches are more likely to be successful and may target one of the major factors that contribute to the development of congestive heart failure. So more clinical trials are needed.

7	Jablonski and Chonchol 2014 [35]	“Recent Advances in the Management of Hemodialysis Patients: A Focus on Cardiovascular Disease”	Morbidity and mortality in maintenance hemodialysis patients are extremely high, and management of care is complex. Treatment options for reducing CVD events, mortality, or both in maintenance hemodialysis are currently limited, and future randomized controlled trials related to each of these points should be considered, with the goal of ultimately improving hard outcomes.

8	Inrig 2013 [36]	“Peri-Dialytic Hypertension and Hypotension: Another U-Shaped BP-Outcome Association”	This study describes the relationship between pre- and postdialysis changes in BP and mortality. The study demonstrated adverse outcomes associated both with large decreases and with any increase in blood pressure pre- to postdialysis.

9	Roberts et al. 2012 [37]	“Challenges in Blood Pressure Measurement in Patients Treated with Maintenance Hemodialysis”	Emerging evidence indicates that standardized hemodialysis unit blood pressure measurements or measurements obtained at home, either by the patient or using an ambulatory blood pressure monitor, may offer advantages over routine hemodialysis unit blood pressure measurements for determining cardiovascular risk and treatment.

10	Altun et al. 2012 [38]	“Prevalence, Awareness, Treatment and Control of Hypertension in Adults with Chronic Kidney Disease in Turkey: Results from the CREDIT Study”	HTN is high risk/awareness to control is essential.

11	Chang 2011 [39]	“Systolic Blood Pressure and Mortality in Patients on Hemodialysis”	The association of SBP with clinical outcomes in patients on hemodialysis is complex. With annual mortality rates for patients on hemodialysis approaching 20% and over half of all these deaths being attributable to cardiovascular causes, future clinical trials that will elucidate ways to improve outcomes for these highest-risk patients are desperately needed.

12	Singapuri and Lea 2010 [40]	“Management of Hypertension in the End-Stage Renal Disease Patient”	Vigorous control of hypertension is recommended.

13	Peixoto and Santos 2010 [41]	“Blood Pressure Management in Hemodialysis: What Have We Learned?”	Until more research is available, we should manage hypertension in hemodialysis with the use of conservative targets, aggressive pursuit of euvolemia through dry weight probing and limitation of sodium overload, and judicious use of medications from conventional drug classes.

14	Malliara 2007 [42]	“The Management of Hypertension in Hemodialysis and CAPD Patients”	The relationship of hypertension with adverse outcomes is uncertain in the hemodialysis population. Whether control of hypertension translates into better outcomes is not known, but collective evidence suggests that hypertension should be controlled in hemodialysis patients.

15	Stidley et al. 2006 [43]	“Changing Relationship of Blood Pressure with Mortality over Time among Hemodialysis Patients”	The relationship between baseline BP and mortality changes over time. High systolic BP (>150 mmHg) was associated with increased mortality among patients who survived >3 years. Low pulse pressure was associated with increased mortality. Mild to moderate systolic hypertension was associated with only modest increases in mortality.

16	Agarwal and Andersen 2006 [44]	“Blood Pressure Recordings within and outside the Clinic and Cardiovascular Events in Chronic Kidney Disease”	Ambulatory BP monitoring in patients with CKD adds to our ability to predict cardiovascular end-points, over and above in-clinic BPs. Risk factors that differentiate hypertension or nondipping appear to confer a cardiovascular risk in CKD.

17	Agarwal et al. 2006 [45]	“Pre- and Postdialysis Blood Pressures Are Imprecise Estimates of Interdialytic Ambulatory Blood Pressure”	Dialysis unit BP measurements are imprecise estimates.

18	Saint-Remy and Krzesinski 2005 [46]	“Optimal Blood Pressure Level and Best Measurement Procedure in Hemodialysis Patients”	Management of BP is necessary in the HD population, first by slow and smooth removal of extracellular volume (dry weight) and thereafter by the use of appropriate antihypertensive medication.

19	Agarwal 2005 [47]	“Hypertension and Survival in Chronic Hemodialysis Patients—Past Lessons and Future Opportunities”	Relationship exists between HTN, CV, and total mortality.

20	Agarwal 2005 [48]	“Hypertension in Chronic Kidney Disease and Dialysis: Pathophysiology and Management”	Confounding variables, such as heart failure, can help explain the U-shaped relationship between BP and total mortality. Well-controlled BP in the presence of poor cardiac function is likely to be associated with high cardiovascular mortality. In contrast, poorly controlled BP with intact cardiac function is expected to be associated with increased mortality. If patients with impaired cardiac function constitute a large part of an observational cohort, a U-shaped relationship between BP and total mortality is seen.

21	Boutitie et al. 2002 [49]	“J-Shaped Relationship between Blood Pressure and Mortality in Hypertensive Patients: New Insights from a Meta-Analysis of Individual-Patient Data”	The increased risk for events observed in patients with low blood pressure was not related to antihypertensive treatment and was not specific to blood pressure-related events. Poor health conditions leading to low blood pressure and an increased risk for death probably explain the J-shaped curve.

22	Mitra et al. 1999 [50]	“What is Hypertension in Chronic Hemodialysis? The Role of Interdialytic Blood Pressure Monitoring”	Hypertension in chronic HD patients contributes significantly to morbidity and mortality. The best representation of interdialytic BP was 20 min postdialysis reading. Walk-in predialysis BP overestimates mean interdialytic BP due to a high incidence of white-coat effect. Ambulatory monitoring has a role in evaluating persistent poor BP control in HD patients.

23	Zager et al. 1998 [17]	““U” Curve Association of Blood Pressure and Mortality in Hemodialysis Patients”	A “U” curve relationship exists between systolic blood pressure and cardiovascular mortality in hemodialysis patients. Multicentered, randomized, controlled clinical trials are required for establishing optimal BP targets in HD patients.

## Additional Points

*Supporting Information*. Refer to S1 PRISMA Checklist, PRISMA 2009 Checklist (DOC).

## References

[1] Fast Facts, The National Kidney Foundation, 2014, https://www.kidney.org/news/newsroom/factsheets/FastFacts

[2] Gilbertson D. T., Liu J., Xue J. L. (2005). Projecting the number of patients with end-stage renal disease in the United States to the year 2015. *Journal of the American Society of Nephrology*.

[3] Lim Y. N., Ong L. M., Goh B. L. (2011). *18th Report of the Malaysian Dialysis and Transplant Registry*.

[4] Orroj H. Chronic Kidney Disease (CKD) challenges in South Asia. http://www.pakistantoday.com.pk/2015/03/03/city/islamabad/chronic-kidney-failure-no-joke-psn/.

[5] US-RDS

[6] Cusumano A. M., Gonzalez Bedat M. C., García-García G. (2010). Latin american dialysis and renal transplant registry: 2008 report (data 2006). *Clinical Nephrology*.

[7] McDonald S., Excell L. (2006). *ANZDATA Registry Report 2005*.

[8] U.S. Renal Data Systems, Annual Data Report, Minneapolis, Minn, USA, 2006

[9] Levey A. S., Beto J. A., Coronado B. E. (1998). Controlling the epidemic of cardiovascular disease in chronic renal disease: what do we know? What do we need to learn? Where do we go from here? National Kidney Foundation Task Force on Cardiovascular Disease. *American Journal of Kidney Diseases*.

[10] Chobanian A. V., Bakris G. L., Black H. R. (2003). Seventh report of the joint national committee on prevention, detection, evaluation, and treatment of high blood pressure. *Hypertension*.

[11] Dahlöf B., Devereux R. B., Kjeldsen S. E. (2002). Cardiovascular morbidity and mortality in the Losartan Intervention For Endpoint reduction in hypertension study (LIFE): a randomised trial against atenolol. *The Lancet*.

[12] Agarwal R., Nissenson A. R., Batlle D., Coyne D. W., Trout J. R., Warnock D. G. (2003). Prevalence, treatment, and control of hypertension in chronic hemodialysis patients in the United States. *The American Journal of Medicine*.

[13] Foley R. N., Herzog C. A., Collins A. J. (2002). Blood pressure and long-term mortality in United States hemodialysis patients: USRDS Waves 3 and 4 Study. *Kidney International*.

[14] Levey A. S., Rocco M. V., Anderson S. (2004). K/DOQI clinical practice guidelines on hypertension and antihypertensive agents in chronic kidney disease. *American Journal of Kidney Diseases*.

[15] National Kidney Foundation (2005). K/DOQI clinical practice guidelines for cardiovascular disease in dialysis patients. *American Journal of Kidney Diseases*.

[16] Foley R. N., Parfrey P. S., Harnett J. D., Kent G. M., Murray D. C., Barre P. E. (1996). Impact of hypertension on cardiomyopathy, morbidity and mortality in end-stage renal disease. *Kidney International*.

[17] Zager P. G., Nikolic J., Brown R. H. (1998). ‘U’ curve association of blood pressure and mortality in hemodialysis patients. *Kidney International*.

[18] Port F. K., Hulbert-Shearon T. E., Wolfe R. A. (1999). Predialysis blood pressure and mortality risk in a national sample of maintenance hemodialysis patients. *American Journal of Kidney Diseases*.

[19] Salem M. M., Bower J. (1996). Hypertension in the hemodialysis population: any relation to one-year survival?. *American Journal of Kidney Diseases*.

[20] Park J., Rhee C. M., Sim J. J. (2013). A comparative effectiveness research study of the change in blood pressure during hemodialysis treatment and survival. *Kidney International*.

[21] Kalantar-Zadeh K., Block G., Humphreys M. H., Kopple J. D. (2003). Reverse epidemiology of cardiovascular risk factors in maintenance dialysis patients. *Kidney International*.

[22] Kalantar-Zadeh K., Kilpatrick R. D., McAllister C. J., Greenland S., Kopple J. D. (2005). Reverse epidemiology of hypertension and cardiovascular death in the hemodialysis population: the 58th annual fall conference and scientific sessions. *Hypertension*.

[23] Gupta N. E. (2007). Reverse epidemiology: dialysis paradox. *Renal and Urology News*.

[24] Chao C.-T., Huang J.-W., Yen C.-J. (2015). Intradialytic hypotension and cardiac remodeling: a vicious cycle. *BioMed Research International*.

[25] Charra B., Chazot C., Jean G. (2003). Reverse epidemiology and hemodialysis blood pressure. *Kidney International*.

[26] Liberati A., Altman D. G., Tetzlaff J. (2009). The PRISMA statement for reporting systematic reviews and meta-analyses of studies that evaluate health care interventions: explanation and elaboration. *Annals of Internal Medicine*.

[27] Moher D., Shamseer L., Clarke M. (2015). Preferred reporting items for systematic review and meta-analysis protocols (PRISMA-P) 2015 statement. *Systematic Reviews*.

[28] Shamseer L., Moher D., Clarke M. (2015). Preferred reporting items for systematic review and meta-analysis protocols (PRISMA-P) 2015: elaboration and explanation. *The British Medical Journal*.

[29] Diaz H. J., Bryan J. M., Arraut J. C., Betancourt J., Cangiano J. L. (2015). Mortality and intradialytic hypertension and hypotension episodes. *Journal of the American Society of Hypertension*.

[30] Myers O. B., Adams C., Rohrscheib M. R. (2010). Age, race, diabetes, blood pressure, and mortality among hemodialysis patients. *Journal of the American Society of Nephrology*.

[31] Gorsane I., Mahfoudhi M., Younsi F., Helal I., Abdallah T. B. (2015). Prevalence and risk factors of hypertension in hemodialysis. *Open Journal of Nephrology*.

[32] Iseki K. (2015). Control of hypertension and survival in haemodialysis patients. *Nephrology*.

[33] Enam N., Kakkad K., Amin A., Lever C. (2014). Management of hypertension in the hemodialysis population: a review of the literature. *Journal of Community Hospital Internal Medicine Perspectives*.

[34] Agarwal R., Flynn J., Pogue V., Rahman M., Reisin E., Weir M. R. (2014). Assessment and management of hypertension in patients on dialysis. *Journal of the American Society of Nephrology*.

[35] Jablonski K. L., Chonchol M. (2014). Recent advances in the management of hemodialysis patients: a focus on cardiovascular disease. *F1000Prime Reports*.

[36] Inrig J. K. (2013). Peri-dialytic hypertension and hypotension: another U-shaped BP-outcome association. *Kidney International*.

[37] Roberts M. A., Pilmore H. L., Tonkin A. M. (2012). Challenges in blood pressure measurement in patients treated with maintenance hemodialysis. *American Journal of Kidney Diseases*.

[38] Altun B., Süleymanlar G., Utaş C. (2012). Prevalence, awareness, treatment and control of hypertension in adults with chronic kidney disease in turkey: results from the CREDIT study. *Kidney and Blood Pressure Research*.

[39] Chang T. I. (2011). Systolic blood pressure and mortality in patients on hemodialysis. *Current Hypertension Reports*.

[40] Singapuri M. S., Lea J. P. (2010). Management of hypertension in the end-stage renal disease patient. *Journal of Clinical Outcomes Management*.

[41] Peixoto A. J., Santos S. F. F. (2010). Blood pressure management in hemodialysis: what have we learned?. *Current Opinion in Nephrology and Hypertension*.

[42] Malliara M. (2007). The management of hypertension in hemodialysis and CAPD patients. *Hippokratia*.

[43] Stidley C. A., Hunt W. C., Tentori F. (2006). Changing relationship of blood pressure with mortality over time among hemodialysis patients. *Journal of the American Society of Nephrology*.

[44] Agarwal R., Andersen M. J. (2006). Blood pressure recordings within and outside the clinic and cardiovascular events in chronic kidney disease. *American Journal of Nephrology*.

[45] Agarwal R., Peixoto A. J., Santos S. F. F., Zoccali C. (2006). Pre- and postdialysis blood pressures are imprecise estimates of interdialytic ambulatory blood pressure. *Clinical Journal of the American Society of Nephrology*.

[46] Saint-Remy A., Krzesinski J.-M. (2005). Optimal blood pressure level and best measurement procedure in hemodialysis patients. *Vascular Health and Risk Management*.

[47] Agarwal R. (2005). Hypertension and survival in chronic hemodialysis patients—past lessons and future opportunities. *Kidney International*.

[48] Agarwal R. (2005). Hypertension in chronic kidney disease and dialysis: pathophysiology and management. *Cardiology Clinics*.

[49] Boutitie F., Gueyffier F., Pocock S., Fagard R., Boissel J. P. (2002). J-shaped relationship between blood pressure and mortality in hypertensive patients: new insights from a meta-analysis of individual-patient data. *Annals of Internal Medicine*.

[50] Mitra S., Chandna S. M., Farrington K. (1999). What is hypertension in chronic haemodialysis? The role of interdialytic blood pressure monitoring. *Nephrology Dialysis Transplantation*.

